# Fully scalable one-pot method for the production of phosphonic graphene derivatives

**DOI:** 10.3762/bjnano.8.111

**Published:** 2017-05-18

**Authors:** Kamila Żelechowska, Marta Prześniak-Welenc, Marcin Łapiński, Izabela Kondratowicz, Tadeusz Miruszewski

**Affiliations:** 1 Faculty of Applied Physics and Mathematics, Gdansk University of Technology, Narutowicza St. 11/12; 80-233 Gdansk, Poland

**Keywords:** functionalized graphene, graphene oxide, one-pot synthesis, phosphonic derivatives, reduced graphene oxide

## Abstract

Graphene oxide was functionalized with simultaneous reduction to produce phosphonated reduced graphene oxide in a novel, fully scalable, one-pot method. The phosphonic derivative of graphene was obtained through the reaction of graphene oxide with phosphorus trichloride in water. The newly synthesized reduced graphene oxide derivative was fully characterized by using spectroscopic methods along with thermal analysis. The morphology of the samples was examined by electron microscopy. The electrical studies revealed that the functionalized graphene derivative behaves in a way similar to chemically or thermally reduced graphene oxide, with an activation energy of 0.014 eV.

## Introduction

Graphene oxide (GO) with its multifunctionality attracts interest in different fields of science. The chemical nature and reactivity of GO have been studied extensively. Many comprehensive reviews concerning GO chemistry can be found in the literature [1–3]. Several models of the structure of GO were proposed and different chemical pathways for its functionalization were established. According to the widely accepted Lerf–Klinowski model, epoxy and hydroxy groups are present on the basal planes of GO, whereas at their edges carboxyl groups are dominant [1–3]. As stated above, GO platelets have chemically reactive oxygen groups, among which carboxylic groups are considered to be the most active ones. The activation of the –COOH group is usually led by treatment with SOCl_2_, followed by subsequent nucleophilic substitution reaction with, i.e., an alcohol or an amine. Alternatively, some coupling agents as *N*,*N*'-dicyclohexylcarbodiimide (DCC) or 1-ethyl-3-(3-dimethylaminopropyl)carbodiimide (EDC) are used for the synthesis of GO amides or esters. Some other known approaches for GO functionalization via carboxylic groups are based on isocyanate or diisocyanate compounds. Many functionalization protocols of GO were described, but only few papers reported the introduction of phosphorous-containing groups into GO planes.

Recently, Kim and Jeon described a ball milling process to efficiently functionalize and exfoliate pristine graphite directly into graphene phosphonic acid. In the first step, graphite is ball-milled for 48 h with red phosphorus to produce a derivative that is edge-functionalized with phosphorus. Then, upon exposure to air moisture, the resultant derivative of graphite is spontaneously oxidized forming phosphonic groups, with simultaneous exfoliation to produce phosphonic acid graphene [4]. However interesting, the described method is time consuming and requires specialized equipment. A multistep procedure for phosphorylating GO and the usage of the latter as proton exchange membranes component was presented in [5]. Phosphorylated GO was synthesized via distillation–precipitation polymerization using dimethyl vinylphosphonate as monomer together with cross-linker and initiator. This approach required the introduction of an intermediate layer prepared by reacting 3-methacryloxypropyltrimethoxysilan with functionalities present on GO. A polymer with phosphonate groups was grafted on the modified GO surface. After a final hydrolysis step, the phosphonates were transformed into phosphonic acids. Eventually, phosphorylated GO was obtained. However, the phosphorous content was comparatively low. The reaction of a triethyl phosphite with an alkyl halide (Arbuzov reaction) was used by Liu et al. for grafting phosphonates to GO [6]. The as-prepared phosphonated GO was successfully applied to adsorb U(VI) from acidic wastewater. As can be seen, only a few examples of graphene derivatives functionalized with phosphonic acid can be found in the literature. The synthesis of phosphonic and bisphosphonic acids, which are widely used as detergents, pharmaceuticals (mainly for osteoporosis treatment), corrosion inhibitors, selective adsorbents and catalysts, is well known [7–9]. One of the most commonly used reactions is the conversion of carboxylic acids into the respective bisphosphonic acids using a mixture of PCl_3_ and H_3_PO_3_ [9]. In this paper, we present a reaction adapted from classical organic chemistry as a convenient route for the functionalization of graphene with phosphonate groups. This one-pot approach requires inexpensive, readily available reagents (only PCl_3_ and water), which coupled with its simplicity makes it attractive for syntheses. Here, GO was functionalized with a simultaneous reduction to produce phosphonated rGO. The spectroscopic and thermogravimetric analysis confirmed the successful functionalization and simultaneous reduction of GO. The electrical studies showed a substantial increase in conductivity after functionalization. The measured energy of activation equal to 0.014 eV is in accordance with literature reports for reduced graphene oxide (rGO).

The promising uses of graphene derivatives in different fields are hindered by complicated and expensive functionalization methods, suitable only for laboratory scale. Effective functionalization by fully scalable, low-cost methods and detailed characterization of the synthesized nanomaterials is the first step towards utilizing the whole spectrum of applications. Functionalization of carbonaceous nanomaterials with phosphonic groups offers great potential for various applications, e.g., in energy conversion and storage [5,10], flame retardation [4,11], catalysis [12], tissue engineering [13], and purification air and water [6,14].

## Results and Discussion

**Figure 1:**
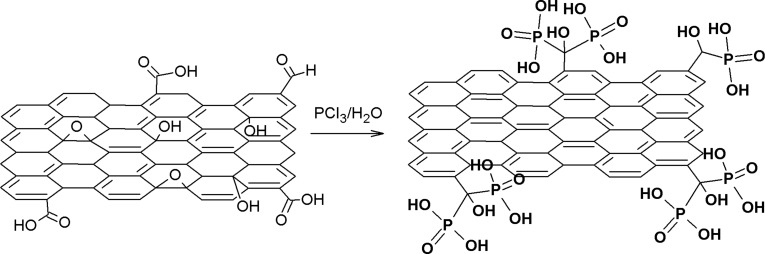
Scheme of GO-P synthesis.

The phosphonic derivative of graphene (GO-P) was obtained through the reaction of GO with phosphorus trichloride in water. Phosphorous trichloride reacts with water to produce phosphorous(III) acid (H_3_PO_3_), so if an excess of PCl_3_ is used, the reaction mixture is eventually PCl_3_/H_3_PO_3_. If GO is introduced into this reaction mixture the carboxylic groups react with PCl_3_ to produce an intermediate product, which is then converted into the respective phosphonate derivative in the reaction with H_3_PO_3_. The scheme of the reaction is presented in Figure 1.

**Figure 2:**
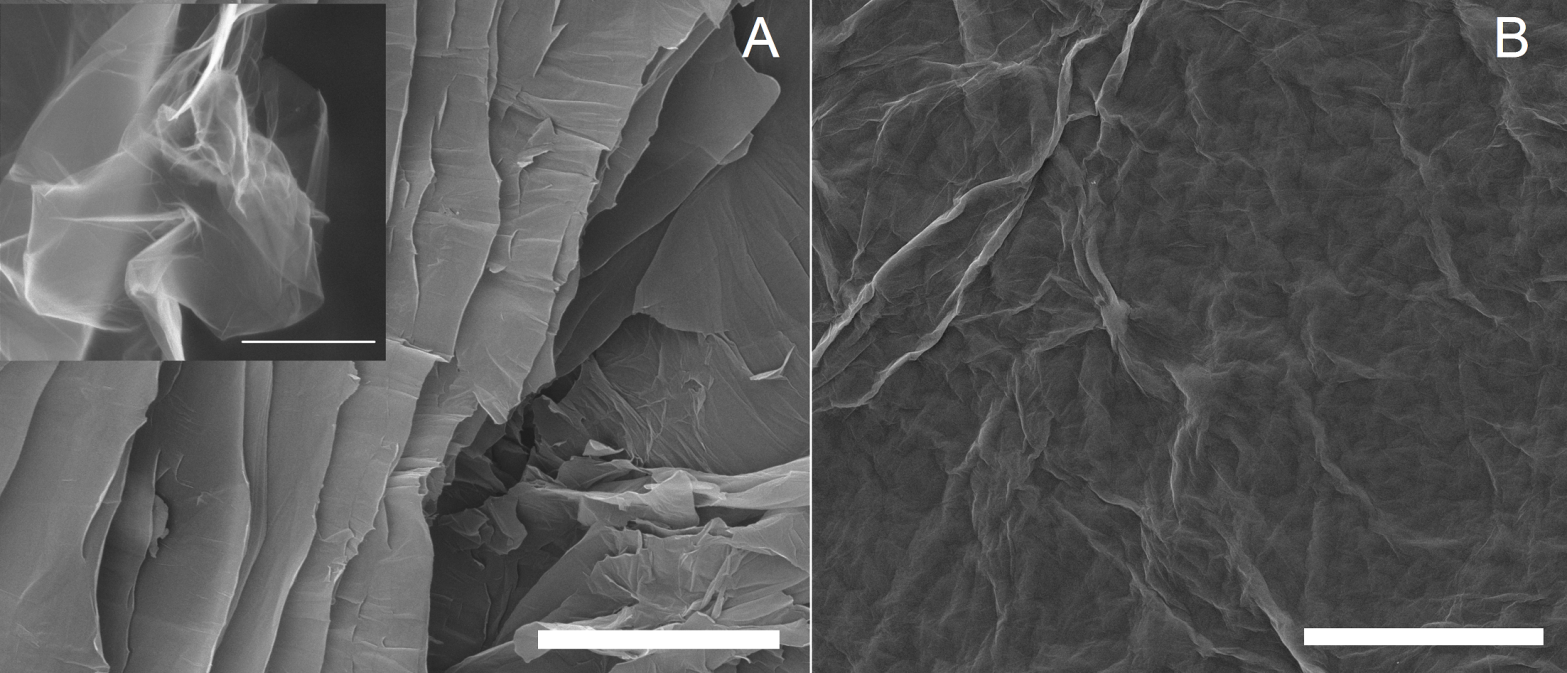
SEM images of A) GO and B) GO-P. The inset in Figure 2A shows a crinkled GO flake. Scale bar: 5 μm.

The first visual observations revealed that after functionalization the color of the previously yellow-brownish GO changed to shiny greyish. The surface morphology of GO and GO-P was examined with SEM (Figure 2). The images demonstrated that GO-P retained a thin-layer morphology similar to that of GO. However, the GO-P sample was less crinkled as most of the functional groups were removed from the basal planes during functionalization.

**Figure 3:**
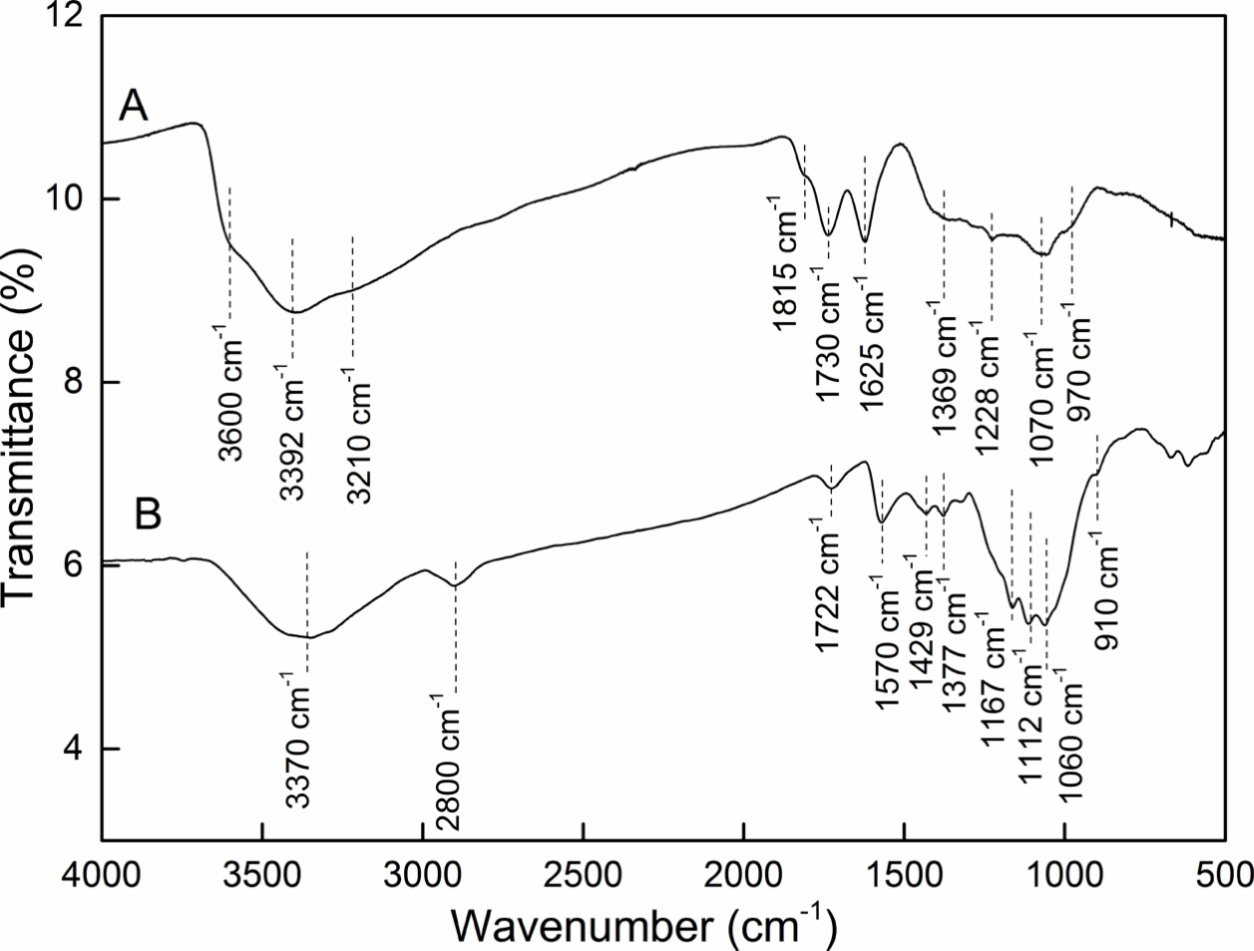
FTIR spectra of A) GO and B) GO-P samples.

Different spectroscopic methods were used to examine the functionalized GO. FTIR spectroscopy was used to determine the type of chemical groups in the analyzed samples. Figure 3 shows FTIR spectra of GO and its phosphonated derivative. In the case of GO the spectrum is in agreement with GO spectra typically observed and reported in the literature [1–3]. A broad band in the region between 3700–3100 cm^−1^ is characteristic for –OH groups of different origin. The band centered at 3600 cm^−1^ comes from the vibrations of free phenolic –OH groups. The next band, at 3392 cm^−1^ with a side band at 3210 cm^−1^ indicates the presence of –OH groups from carboxylic groups, along with –OH from adsorbed water molecules due to high hydrophilicity of the GO. The well-developed band at 1730 cm^−1^ confirms the presence of carboxylic groups in the GO. Stretching vibrations of double carbon–carbon bonds in the GO structure gave the expected band at 1625 cm^−1^. The position of the band indicates that such bonds are conjugated with C=C or C=O bonds. Small bands at 1815 cm^−1^ and 1369 cm^−1^ are characteristic for C=O and C–O stretchings in lactones, respectively. Other bands at lower wavenumbers (1228–970 cm^−1^) can be ascribed to C-O vibrations in carboxyl, phenol and/or epoxide functionalities.

Significant differences can be observed in the spectrum of GO-P. Firstly, the bands in the region of carbonyls vibrations (1815 and 1730 cm^−1^), which were well visible in the GO spectrum, disappeared in the GO-P spectrum. The small band visible in the GO-P spectrum in this region (ca. 1722 cm^−1^) may be due to the –OH vibrations in O=P(OH)_2_, which are of low intensity. Other bands at 1570 cm^−1^ and 1429 cm^−1^ can be ascribed to aromatic C–C bonds. Intensive bands at 1167, 1112 and 1060 cm^−1^ coming from P=O and two P–OH, respectively, confirm the presence of phosphonate groups in the analyzed sample [4–6]. The broad band centered at 3370 cm^−1^ refers to –OH groups in adsorbed water. The new broad and weak band at 2800 cm^−1^ additionally gives evidence that phosphonate groups tend to form hydrogen bonds (within themselves or water molecules) as this band it connected with associated PO–H stretching.

**Figure 4:**
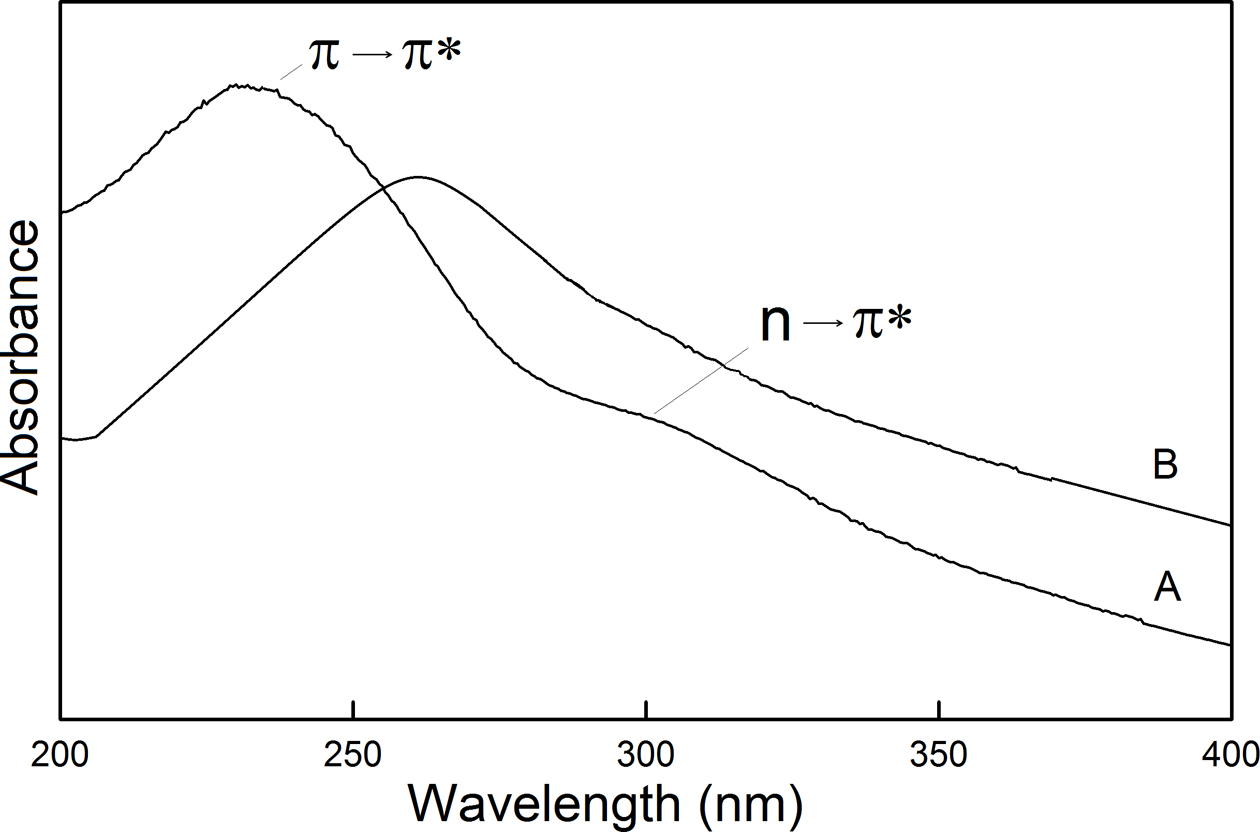
UV–vis spectrum of A) GO sample and B) GO-P sample.

FTIR analysis showed that GO contains carboxyl or carbonyl functional groups connected with C=C bonds, which are also known to absorb light in the UV range. Thus, the UV–vis spectroscopy can be used to monitor structural changes of GO upon functionalization and/or reduction [15–17]. The UV–vis spectra of aqueous suspensions of GO and GO-P are presented in Figure 4. The main maximum absorption band of the GO spectrum (Figure 4A) observed at 230 nm can be ascribed to π→π* electron transitions in conjugated carbon–carbon double bonds in the GO plane. The second, smaller band at 310 nm is connected with n→π* transitions of nonbonding electrons in oxygen atoms connected with C=C bonds. Upon functionalization oxygen functionalities are removed from the basal plane and the structure of conjugated double bonds is restored. Therefore, the small band at 310 nm connected with n→π* electron transition disappeared in the GO-P spectrum. Moreover, the band at 230 nm is red-shifted to 260 nm. This is consistent with the general rule that the addition of conjugated bonds into the structure causes a bathochromic shift of the band position in the UV–vis spectrum. As was mentioned earlier, after functionalization the hexagonal carbon network was restored, so the overall number of conjugated bonds was increased.

**Figure 5:**
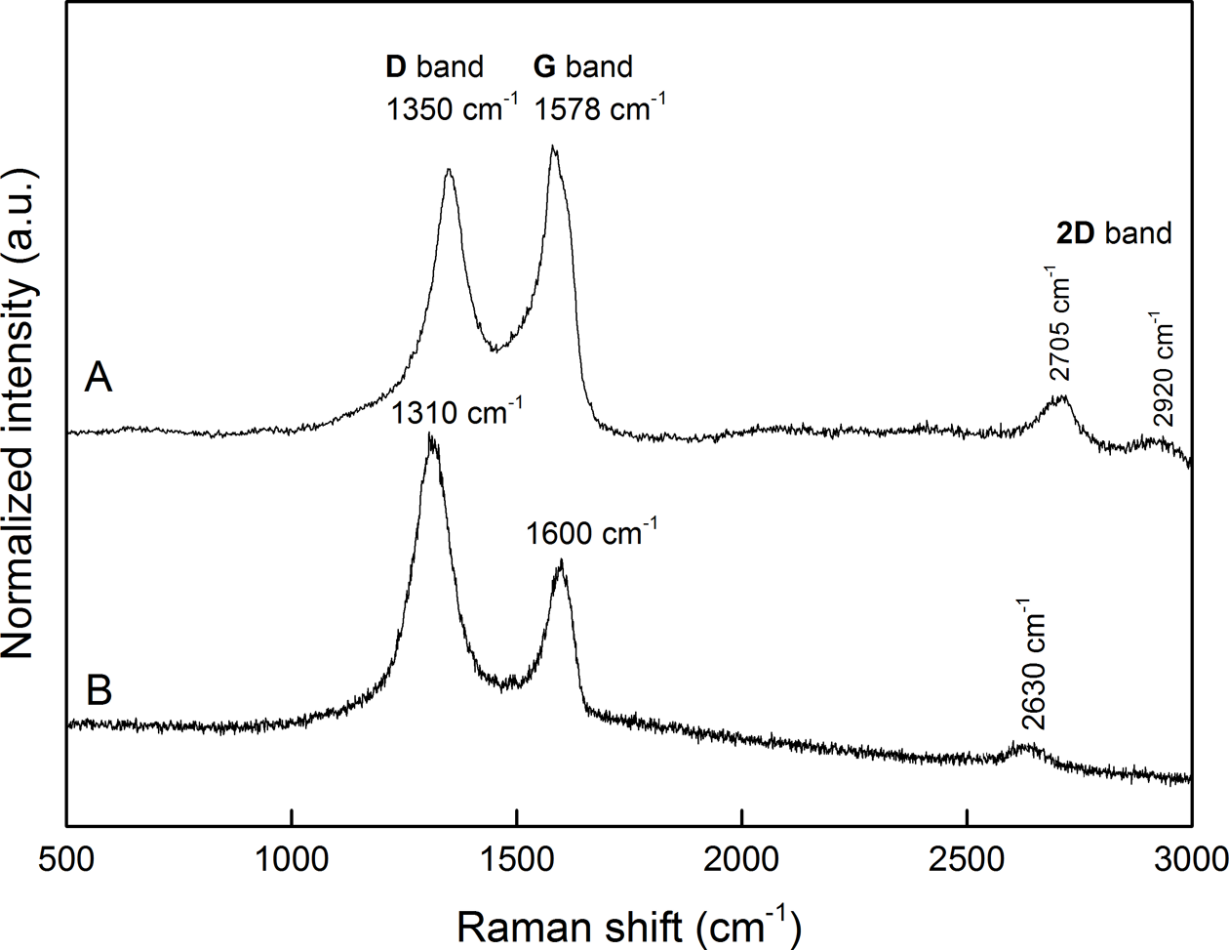
Raman spectra of A) GO and B) GO-P.

Raman spectroscopy is very frequently used to characterize carbonaceous materials including GO and its derivatives. The Raman spectrum of GO (Figure 5A) consists of two main bands: the G band corresponding to the first-order scattering of the E_2g_ mode at 1578 cm^−1^ and broad D band at 1350 cm^−1^. The overtone 2D band can be seen in the range from 2700 to 3000 cm^−1^. The D band is attributed to phonon branches around K and together with its overtone 2D band both are dispersive bands observed in graphite-derived carbon materials. The Raman spectrum of GO-P (Figure 5B) is similar to the spectrum of its precursor, but showing a higher intensity of the D band compared to the G band. A shift of ca. 30 cm^−1^ to lower wavenumbers can be observed for the D band and the G band is shifted to higher wavenumbers by about 20 cm^−1^. A small shift of the 2D peak, together with a decrease of its intensity can also be observed. According to the literature the frequency shift of G and 2D is connected with changes in the number of stacking layers in GO and rGO. It was proved that if the number of layers decreases, the G band position is shifted to higher frequencies. Moreover, a G band shift to higher frequencies was also reported for smaller particles of graphene-type materials, compared to the bigger particles of the same type. Taking this into account, it can be concluded that in the GO-P sample the average size and the number of stacking layers of graphene platelets is smaller than in GO. The Raman spectra of GO and rGO were studied by different groups and there is some inconsistency regarding their interpretation. More often, the *I*_D_/*I*_G_ ratio before and after reduction of GO is compared and changes are ascribed to a successful reduction. More recent studies revealed, however, that this approach is without scientific value. The analysis of G and 2D bands gives more insight into the structure of graphene derivatives. According to the literature a decrease of the 2D band intensity with respect to the G band intensity is a proof of GO reduction [18–20]. *I*_2D_/*I*_G_ ratios of 0.12 for GO and of 0.025 for GO-P confirms the reduction of GO. Moreover, the combined peak in the GO spectrum at 2920 cm^−1^, which requires defects for its activation, disappeared in the GO-P sample. It also proves, that after functionalization the conjugated bonds in the graphene plane were restored, and functional groups are present mainly at the edges.

**Figure 6:**
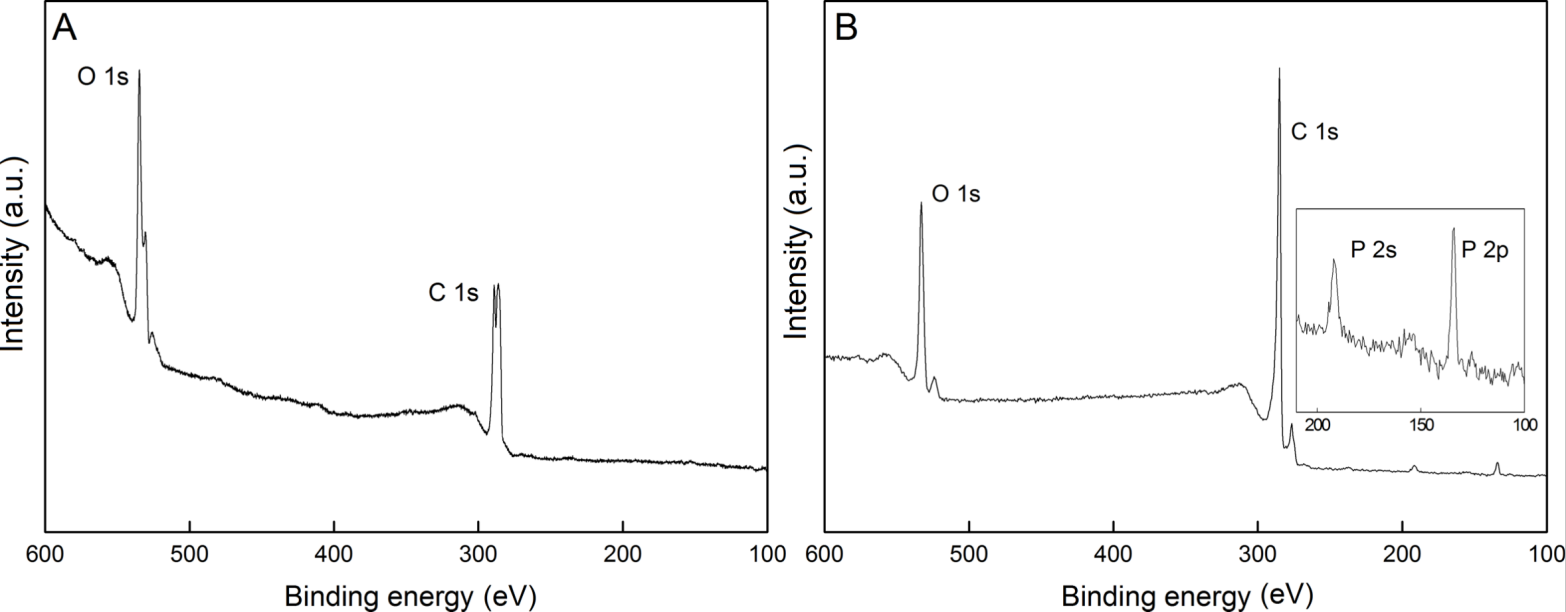
XPS spectra of A) GO and B) GO-P. The inset shows an enlarged view of the energy region characteristic for P.

**Figure 7:**
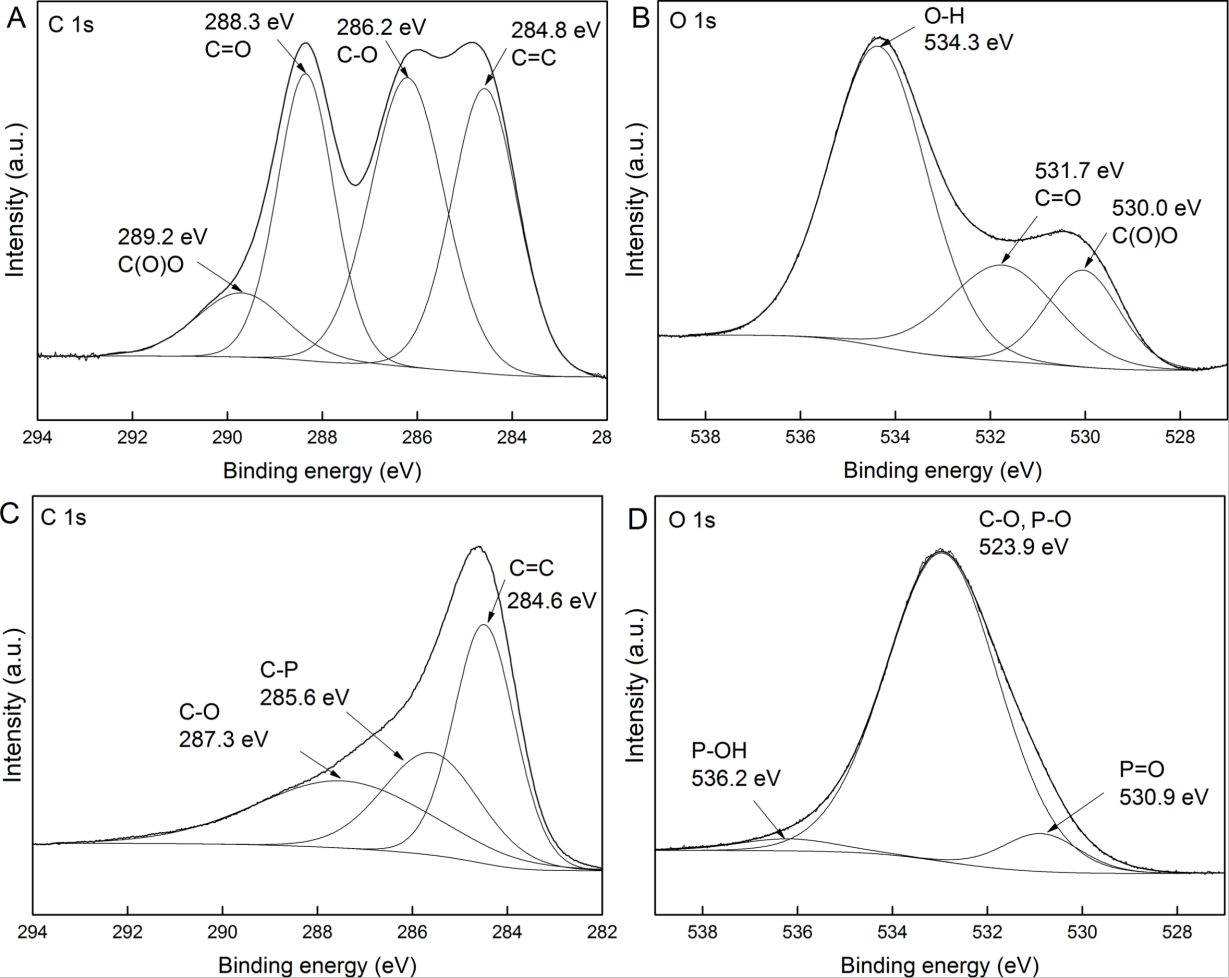
High-resolution XPS spectra with curve fittings for the C 1s and O 1s for GO (A and B) and GO-P (C and D).

X-ray photoelectron spectroscopy (XPS) further confirmed the presence of phosphorous in the GO-P sample. The XPS spectra of GO and GO-P are presented in Figure 6. The initial GO (Figure 6A) showed prominent O 1s and C 1s peaks at 534.3 and 285.5 eV, respectively. In the case of GO-P (Figure 6B), in addition to the C 1s (at 285.0 eV) and O 1s (at 533.0 eV) peaks, new peaks at 191.5 eV and 135.7 eV were observed and assigned as P 2s and P 2p peaks (see inset Figure 6B). The content of phosphorus was calculated to be 2.15%. High-resolution XPS spectra of GO with curve fittings for C 1s (Figure 7A) and O 1s (Figure 7B) clearly demonstrate the high degree of GO functionalization. The multi-band spectra of O 1s, as well as C 1s, indicate a variety of oxygen functionalities in the analyzed sample, including hydroxy, carboxyl and epoxide groups. In the case of the GO-P sample, the peaks referring to covalent carbon–oxygen bonds are reduced in their intensity and new peaks corresponding to covalent C–P and P–O bonds were denoted. The C 1s spectrum of the GO–P sample is very similar to the spectra reported for thermally or chemically reduced GO [21–23], but with an additional peak ascribed to C–P bonds. Moreover, the O 1s peaks showed that P–O and P=O are the dominant P components in GO-P.

The high P content, together with the simplicity of the reaction makes the proposed method attractive compared to previously published protocols. For example, Liu et al. [6] reported an Arbuzov reaction to functionalize GO and calculated the content of P-bearing groups to be 2.37%. An Arbuzov reaction uses phosphonic acid esters and an hydrolysis step is necessary to obtain phosphonic acids, which in the case of phosphonate esters requires harsh conditions. In the phosphorylated GO nanosheets reported by Bai et al. the weight percentage of P was about 0.5% [5], which is unacceptably low taking into account the multi-step approach. According to Kim et al. in phosphorous-doped graphene obtained by ball-milling the loading amount of phosphorus could reach up to 23.9 wt % [4]. However, this method suffers from several impediments. First of all the reaction is carried out in a ball miller in vacuum for days. The purification process is also burdensome, as the final product is Soxhlet-extracted with PBr_3_, producing hazardous waste. Finally, after ball milling for two days the resultant carbonaceous material is rather high-surface-area graphite than graphene.

**Figure 8:**
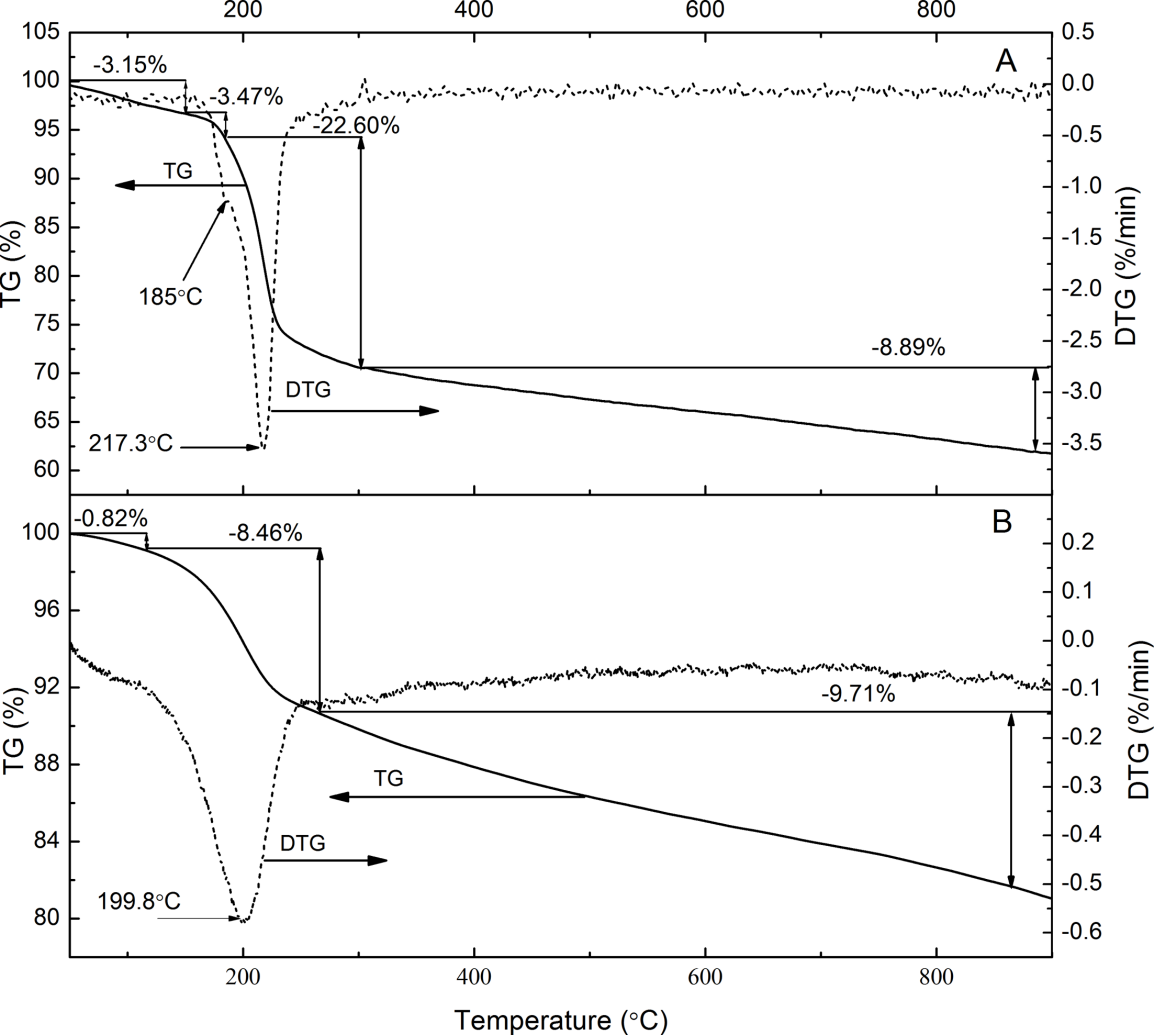
TG and DTG curves of A) GO and B) GO-P.

Thermogravimetric (TG) analysis with differential scanning calorimetry (DSC) provided information about thermal stability of GO and GO-P and allowed for an estimation of the functionalization degree. As can be seen in Figure 8A, GO showed a total mass loss of ca. 40%. Water desorption (ca. 3.5%) was observed below 150 °C and the subsequent rapid mass loss in the range of 150–300 °C is connected to the detachment of oxygen functional groups. The TG curve of GO-P (Figure 8B) revealed a lower water desorption (ca. 0.8%) and the total mass loss was also lower with the residual mass being about 80% of the initial mass. The TG curve profile is different for both samples, showing different steps between 150 and 300 °C, confirming the presence of different functional groups. The differences are well noticeable in the DTG curves. For the GO sample two local minima at 185.0 and 217.3 °C were observed, while the GO-P sample revealed one broad minimum at 199.8 °C. The lower mass loss observed for the GO-P compared to its precursor is reasonable and is in agreement with other results presented in this paper. During the synthesis, carboxylic groups are converted into phosphonate groups and most of the other groups (epoxy or hydroxy) are removed from the graphene plane. So, GO-P is a reduced GO with functional groups located mainly at its edges. The mass loss in the temperature range of 300–900 °C is similar for both samples, showing that after detachment of functional groups a similar decomposition of carbonaceous material occurs.

**Figure 9:**
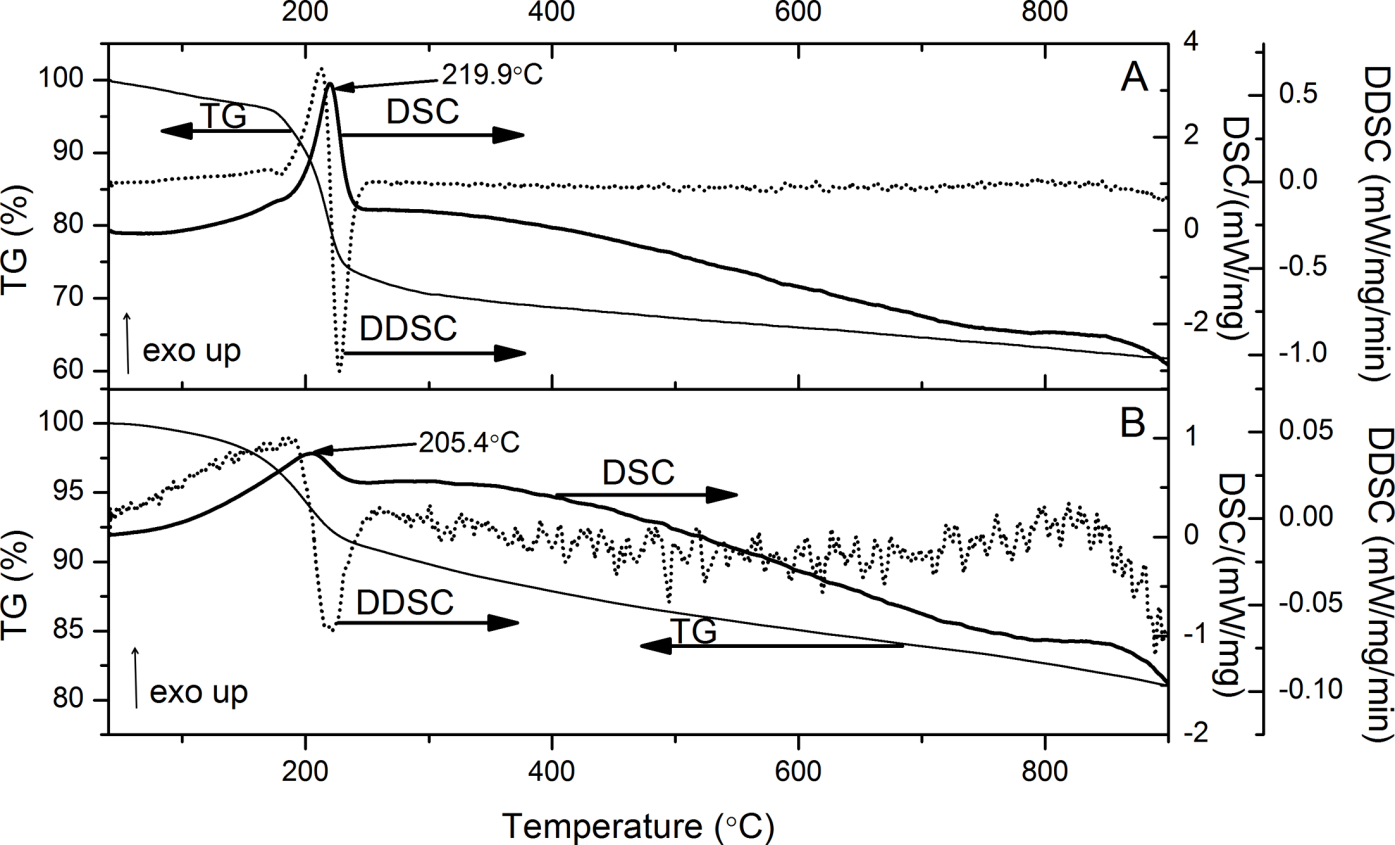
DSC and DDSC curves of A) GO sample and B) GO-P. The TG curves are the same as in Figure 8.

The DSC analysis also revealed differences in thermal properties of the studied samples. The DSC profile of GO and functionalized GO is not frequently reported. Figure 9 shows the DSC and the DDSC (derivative of DSC) curves for both samples. This is the first time that a phosphonated graphene derivative is studied by DSC. For comparison, the DSC results of the GO sample are shown (Figure 9A). It should be noted that for both samples the peaks observed in DSC curve correspond to the mass loss in the TG curve. The exothermic peak, observed for both samples near 200 °C is connected to the decomposition of the analyzed material. The heat released during the decomposition of the GO-P sample is much smaller than that of GO. This is advantageous for the usage of GO-P as flame retardant, similarly as was reported by Kim et al. [4].

**Figure 10:**
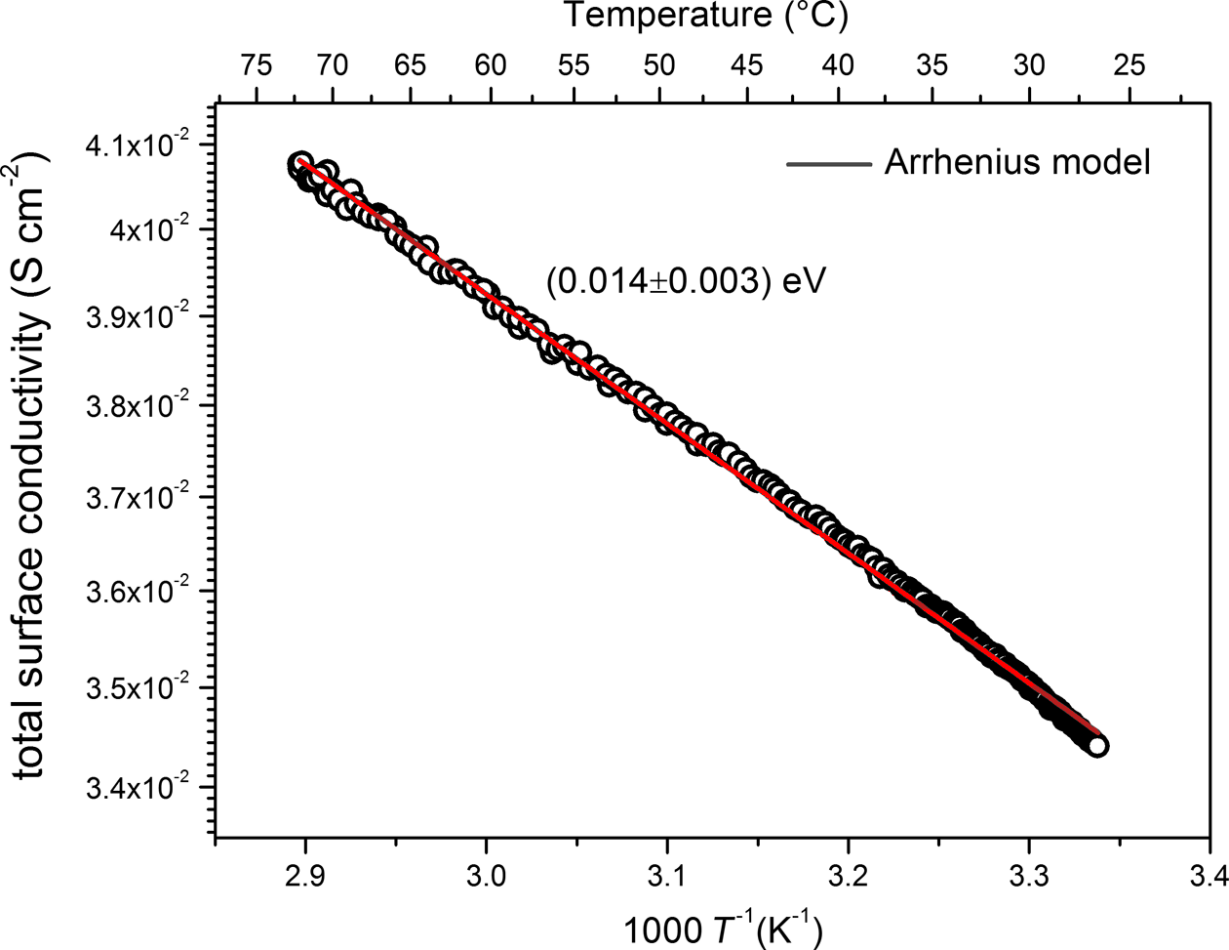
The temperature dependence of the total surface conductivity of the analyzed GO-P sample.

Finally, a measurement of the electrical properties of GO-P materials was carried out. Before reduction, the GO layers exhibited insulating behavior, with a resistivity higher than 10^6^ Ω·cm. The temperature-dependent electrical conductivity (σ) of GO-P was investigated in a temperature range from 25 to 70 °C. The functionalization with simultaneous reduction resulted in a pronounced increase of conductivity compared to pristine GO (Figure 10). As can be seen, the surface conductivity reaches high values (3.4 × 10^−2^ S·cm^−2^ to 4.2 × 10^−2^ S·cm^−2^ in the measured temperature range) and increases with increasing temperature. Moreover, an exponential temperature dependence of conductivity was noticed. The red line presented in Figure 10 represents the fit of an Arrhenius model (σ ~ exp(−T^−1^)). The good correlation between applied model and experimental results (R^2^ ≈ 0.998) indicates that the electrical conductivity at higher temperatures of the analyzed GO-P sample can be well described by an Arrhenius temperature dependence model for semiconductors.

The results presented in Figure 10 clearly shows that the phosphonated graphene derivative (GO-P) behaves in a way similar to that of reduced GO. This suggests that the functional groups are mainly connected to carbon atoms located at the edges of nanostructure, and the basal plane was restored after functionalization. Thus, the synthesis method presented in this paper allows to obtain the graphene-type material with comparable electrical properties without additional reduction processes, such as thermal reduction. Moreover, the activation energy calculated from the slope of the plotted line in Figure 10 is equal to 0.014 eV. The reported values for different graphene derivatives cover a wide range between 0.005 and 0.730 eV [24–28]. It is known that different reducing methods are used to reduce GO into rGO and, depending on the quality of the initial GO and the reducing approach, the properties of obtained rGO may vary [1–3,24–28].

## Conclusion

The development of industrially applicable methods for the production of graphene derivatives will pave the way for a wider spectrum of applications. In this paper, a simple one-pot synthesis approach for the functionalization of a graphene derivative was presented. The method benefits from the easiness, fastness, the usage of inexpensive and readily available chemicals and it is fully scalable. The resultant phosphonated graphene was demonstrated to form stable suspensions in various polar solvents including water, which is the preferable solvent for numerous applications. The obtained material was fully characterized and the obtained results confirmed the successful functionalization with simultaneous reduction of GO. FTIR and XPS spectroscopy confirmed the successful introduction of phosphonate groups into GO-P. The phosphorous content calculated from XPS results was 2.15%. Raman spectroscopy, along with UV–vis and XPS spectroscopy results proved the simultaneous restoration of conjugated double bonds in the graphene plane. The functionalized material revealed an electrical conductivity consistent with values reported for chemically or thermally reduced GO. The conducting graphene derivative with polar multiprotic phosphonate groups can find its place in, for example, conducting composites, ion exchangers and proton carriers.

## Experimental

### Chemicals

PCl_3_ was purchased from POCh (Gliwice, Poland). Spectral grade KBr was purchased from Sigma-Aldrich. Deionized water was used in the experiments.

### Apparatus

During the experiments a magnetic stirrer Heidolph MR hei-standard with hot plate was used and the centrifugation for all samples was done using a Chemland model P3032 centrifuge at a speed 15000 rpm for 10 min.

The FTIR spectra were recorded using the KBr pellet method on a Perkin Elmer Frontier spectrophotometer with resolution of 2 cm^−1^ in the range of 500–4000 cm^−1^.

UV–vis spectra were measured using a Perkin Elmer Lambda 10 with 1 cm quartz cuvette.

Raman spectra were recorded using a Renishaw InVia spectroscope with argon ion laser operating at 514.5 nm focused through a 50× objective. The collected light was dispersed through a triple monochromator and detected with a charge-coupled device. The spectra were collected in the dark, with a resolution of 2 cm^−1^ in the range of 100–3200 cm^−1^.

The morphology of the samples was observed with a scanning electron microscopy (ESEM Quanta Feg 250, FEI).

XPS analyses were carried out with an X-ray photoelectron spectrometer (Omicron NanoTechnology) with 128-channel collector. The measurements were performed with constant energy mode (CAE) with energy pass equal to 50 eV. The samples were pressed before analysis. XPS measurements were performed at room temperature under ultra-high vacuum conditions, below 1.1 × 10^−8^ mbar. The photoelectrons were excited with an Mg Kα X-ray source. The X-ray anode was operated at 15 keV and 300 W. An Omicron Argus hemispherical electron analyser with round aperture of 4 mm was used for analyzing the emitted photoelectrons. The binding energies were corrected using the background C 1s line (285.0 eV) as a reference [29]. XPS spectra were analysed with Casa-XPS software using a Shirley background subtraction and Gaussian–Lorentzian fits.

Simultaneous thermogravimetric analysis (TGA) and differential scanning calorimetry (DSC) were performed in synthetic air with a heating rate of 5 °C/min from 40 to 900 °C using a Netzsch STA 449 F1. The STA449 F1 has a vertical sample carrier and in order to account for buoyancy effects, a correction curve with empty crucibles was first obtained and then subtracted from the experimental results. To avoid heat and mass transfer limitations, approximately 8 × 10^−6^ kg of sample was used, and Al_2_O_3_ crucibles with lids were employed. The total uncertainty associated with measurement was 0.005% by weight of the sample and was included in the final result. The first derivatives of TGA and DSC, denoted as DTG and DDSC, respectively, were calculated using Netzsch Proteus Thermal Analysis program.

In order to investigate the electrical properties of obtained GO structures, the total surface conductivity (σ) was measured by a conventional DC four-wire method with a Keysight 34970A multimeter. The measurements were performed in a temperature range of 25–70 °C in air atmosphere. The sample was prepared as a thin film of rectangular shape.

### Synthesis

GO was synthesized by using the improved Hummer’s method proposed by Tour’s group and fully characterized as described previously [11–12]. To a paste of GO in water (50 mg/mL), the ca. three-fold excess of PCl_3_ was added dropwise under simultaneous agitation. After 5 h of heating at 65 °C, the excess of PCl_3_ was distilled off and the residue was diluted with water and stirred for 1 h at 50 °C. Then, the solids were separated by centrifugation, washed with deionized water till a neutral pH value and dried overnight under reduced pressure (30 °C, 0.01 bar) to obtain GO-P.

## Supporting Information

File 1 Additional experimental data.
