# Supplementary material for: Fully scalable one-pot method for the production of phosphonic graphene derivatives
Source: Beilstein J Nanotechnol. 2017 May 18;8:1094–103. doi: 10.3762/bjnano.8.111 (PMC5480340; doi:10.3762/bjnano.8.111)
Supplement: File 1 — Additional experimental data. [file Beilstein_J_Nanotechnol-08-1094-s001.pdf]

Supporting Information

for

Fully scalable one-pot method for the production of phosphonic graphene derivatives

Kamila Żelechowska*, Marta Prześniak-Welenc, Marcin Łapiński, Izabela Kondratowicz and Tadeusz Miruszewski

Address: Faculty of Applied Physics and Mathematics, Gdansk University of Technology, Narutowicza St. 11/12; 80-233 Gdansk, Poland.

Email: Kamila Żelechowska* - kzelechowska@mif.pg.gda.pl

* Corresponding author

Additional experimental data

1. Mechanism of the reaction

The reported by us chemical functionalization of GO was inspired by the reaction used in the 1-hydroxyethylidene-1,1-bisphosphonic acids synthesis. 1-Hydroxyethylidene-1,1-bisphosphonic acids are perhaps the oldest group of bisphosphonates. They are commonly prepared by a large-scale, one-step reaction of carboxylic acids with phosphorus trichloride and phosphorous or phosphoric acids, followed by hydrolysis with water. The reaction is carried out in selected solvents (phenylsulphonic acid, various phenols, chlorobenzene, diphenyl ether or ionic liquids) with sulfone and methanesulphonic acid being preferred choices. The mechanism of the reaction, reported elsewhere [1,2] is shown in Figure S1. The formation of carboxylic acid chloride as a first intermediate has been demonstrated (Figure S1. Scheme 1, compound I). It was proved, that simultaneously the stepwise hydroxylation of phosphorus trichloride occurs (Figure S1. Scheme 2), producing the intermediates $P(OH)Cl_2$ and $P(OH)_2Cl$ (Figure S1. Scheme 2, compound II), which may react with other molecules of carboxylic chloride (Figure S1, Scheme 4) or degrade to hydrogen chloride and condensed phosphorus acids (Figure S1. Scheme 3) [2]. The intermediate trivalent P-OH species react with carboxylic acid chloride (Figure S1, Scheme 4). The formed ketophosphonate (Figure S1, Scheme 4, compound III) can further react with trivalent P-OH. Bisphosphonic derivative (Figure S1. Scheme 4, compound IV) is obtained after hydrolysis. It has also been documented that the use of phosphorous acid could be omitted if water was added to the reaction medium, and thus this compound is formed in situ [2]. Experience has shown that the reaction of carboxylic acids with H_3PO_3 without addition of PCl_3 does not take place. In our approach, we eliminated the usage of solvent and used only PCl_3 and water, which served as reactants and reaction medium.

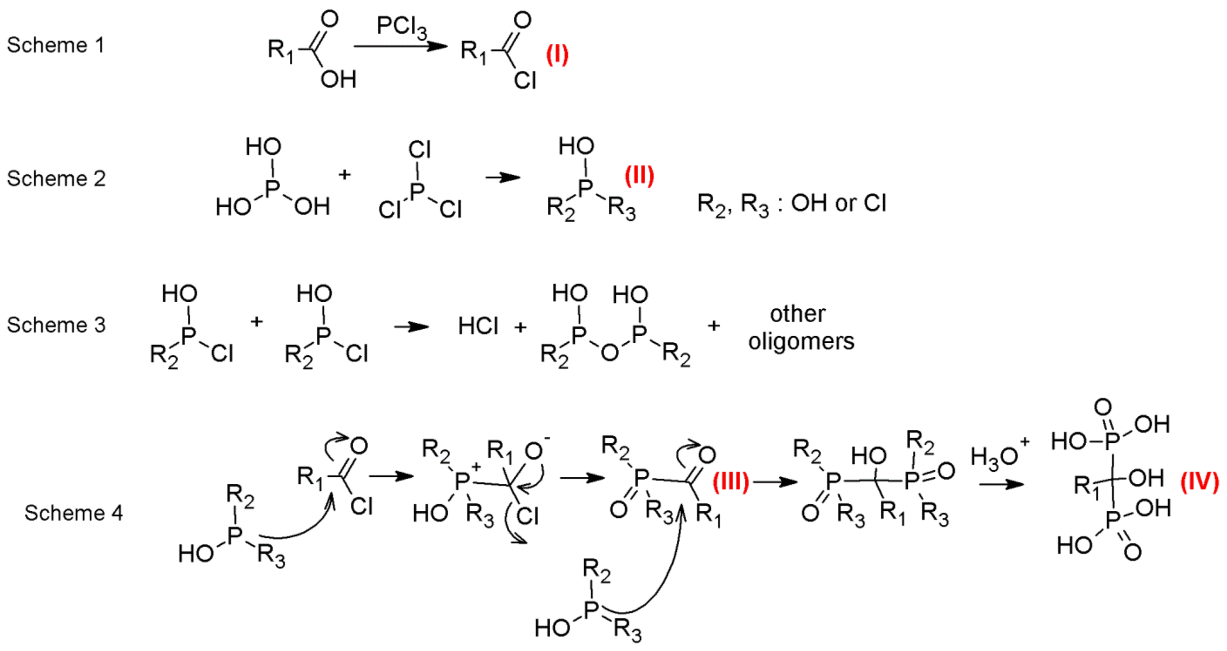

Figure S1: Mechanism of carboxylic acids conversion into bisphosphonic derivatives.

2. Visual changes of the GO after functionalization

GO, depending on the flake size and oxygen groups content is yellow or brownish. After functionalization, obtained GO-P was grayish or black, resembling rGO obtained by chemical reduction of GO. In Figure S2 the photographs of GO (A) and GO-P (B) as prepared can be seen.

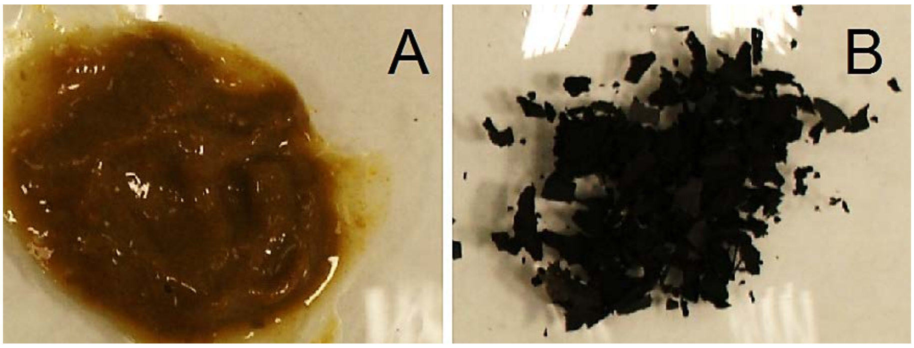

Figure S2: Photograph of GO (A) and GO-P (B).

If the functionalization reaction was performed on the dry thin film of GO, the greyish paper-like GO-P was produced (see Figure S3).

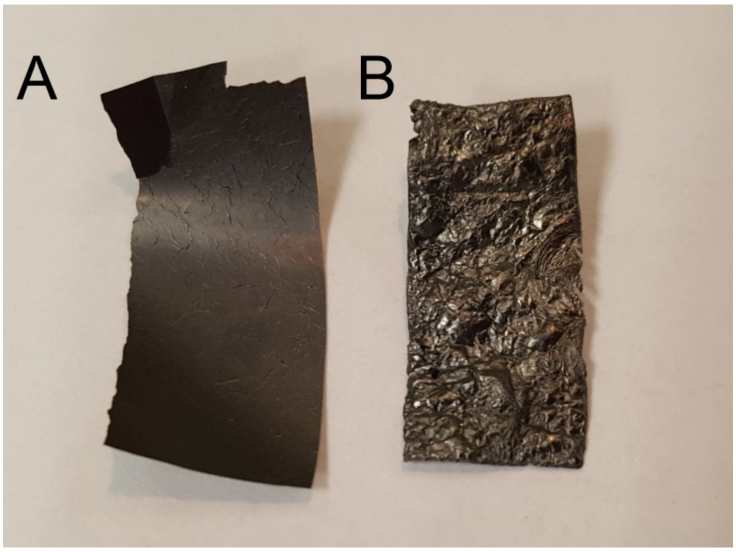

Figure S3: Photograph of paper-like (GO) and (GO-P).

3. X-ray powder diffraction spectroscopy

XRD measurements were carried out to analyze the layer-to-layer distance (d-spacing) between graphene sheets and the main crystalline size (that is a height of the stacked layers) in GO-P. The Bragg's equation was applied to calculate the distance (d) between layers in GO-P sample. Scherrer's equation with a constant k = 0.9 was applied for evaluating the average height of stacked layers, denoted as H. The number of stacked graphene layers was also evaluated. XRD measurements were performed using Philips X'PERT PLUS diffractometer with Cu Kα radiation (λ = 0.154 nm).

As compared to the XRD spectrum of GO (Figure S4), the removal of functional groups led to the decrease in the interlayer spacing, from 0.863 nm for GO to 0.365 nm for GO-P and it was slightly higher than that of graphite (0.336 nm). This suggests that some functional groups are present on planes and edges of graphene. The broad XRD signals of the GO indicated the poor ordering of graphene sheets along their stacking direction. The height of stacking GO layers was found to be 15.12 nm and decreased to 2.96 nm for GO-P. The number of layers was calculated to be 17 for pristine GO and decreased to 5–8 layers for GO-P.

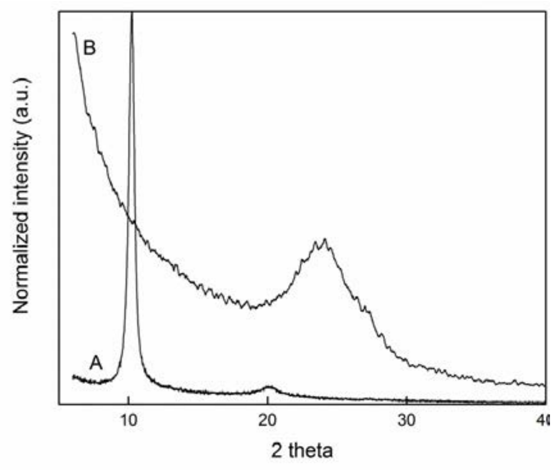

Figure S4: XRD spectra of GO (A) and GO-P (B).
